# Geospatial Analysis and Seasonal Distribution of West Nile Virus Vectors (Diptera: Culicidae) in Southern Ontario, Canada

**DOI:** 10.3390/ijerph15040614

**Published:** 2018-03-28

**Authors:** Bryan V. Giordano, Kevin W. Turner, Fiona F. Hunter

**Affiliations:** 1 Centre for Biotechnology, Brock University, St. Catharines, ON L2S 3A1, Canada; bg08ts@brocku.ca; 2 Centre for Vector-Borne Disease, Brock University, St. Catharines, ON L2S 3A1, Canada; 3 Department of Geography and Tourism Studies, Brock University, St. Catharines, ON L2S 3A1, Canada; kturner2@brocku.ca; 4 Department of Biological Sciences, Brock University, St. Catharines, ON L2S 3A1, Canada; 5 Entomogen Inc., St. Catharines, ON L2R 2N6, Canada

**Keywords:** West Nile virus, Ontario, Canada, mosquito, biogeography, vector, *Aedes*, *Anopheles*, *Culex*, *Ochlerotatus*

## Abstract

The purpose of this study was to establish geospatial and seasonal distributions of West Nile virus vectors in southern Ontario, Canada using historical surveillance data from 2002 to 2014. We set out to produce mosquito abundance prediction surfaces for each of Ontario’s thirteen West Nile virus vectors. We also set out to determine whether elevation and proximity to conservation areas and provincial parks, wetlands, and population centres could be used to improve our model. Our results indicated that the data sets for *Anopheles quadrimaculatus*, *Anopheles punctipennis*, *Anopheles walkeri*, *Culex salinarius*, *Culex tarsalis*, *Ochlerotatus stimulans*, and *Ochlerotatus triseriatus* were not suitable for geospatial modelling because they are randomly distributed throughout Ontario. Spatial prediction surfaces were created for *Aedes japonicus* and proximity to wetlands, *Aedes vexans* and proximity to population centres, *Culex pipiens*/*restuans* and proximity to population centres, *Ochlerotatus canadensis* and elevation, and *Ochlerotatus trivittatus* and proximity to population centres using kriging. Seasonal distributions are presented for all thirteen species. We have identified both when and where vector species are most abundant in southern Ontario. These data have the potential to contribute to a more efficient and focused larvicide program and West Nile virus awareness campaigns.

## 1. Introduction

West Nile virus (WNV; family Flaviviridae, genus *Flavivirus*) has been endemic in Canada for over a decade and continues to be a prominent public health concern for Canadians. It has been estimated that WNV cost the American economy between $700 million and $1 billion from 1999 to 2012 [1]. The economic loss estimates for the United States were based on 37,088 reported cases of WNV over 13 years [2]. By extrapolation, the 5465 Canadian human cases (both endemic and acquired during travel) reported to the Public Health Agency of Canada (PHAC) from 2002 to 2013 in Canada [3] would represent approximately $25 to $275 million in economic losses. This estimate does not include yearly budgets for mosquito control, surveillance programs, or costs for long and short-term disability.

Since the arrival of WNV in 2001, the province of Ontario, Canada has seen an increase in the amount of mosquito surveillance that has been conducted to warn the public of WNV activity. These data are crucial for monitoring arbovirus transmission and the spread of invasive mosquito species. A recent survey of the published literature and surveillance databases has identified that 67 mosquito species are known to inhabit Ontario [4]. Fortuitously, not all mosquito species are capable of transmitting WNV. For human transmission to occur a mosquito must first blood-feed on a WNV-infected bird. The WNV virions from the infected blood-meal must replicate in the mosquito’s mid-gut epithelia, pass into the hemolymph, disseminate to the salivary glands, and accumulate in the saliva secretions [5,6]. West Nile virus is involved primarily in an enzootic cycle involving avian hosts and mosquitoes of genus *Culex* [7,8,9]. Opportunistic species and species with wide-host ranges from other genera such as *Aedes*, *Anopheles*, *Culiseta*, and *Ochlerotatus* have also tested positive for presence of WNV in field-collected specimens [7,9,10,11], which suggests that non-ornithophilic mosquito species also play a role as bridge vectors in the transmission of WNV.

During the initial years of WNV surveillance in Ontario all collected species were identified and tested for presence of WNV to establish which species were involved in WNV transmission. Based on these data, Public Health Ontario (PHO), the governing body of each municipal Public Health Unit (HU), and PHAC have identified thirteen species as implicated in the transmission of WNV in Ontario; these species are referred to as WNV vectors. At the top of the list are *Culex pipiens* Linnaeus, *Culex restuans* Theobald, *Culex salinarius* Coquillett, *Aedes japonicus* (Theobald), *Culex tarsalis* Coquillett, *Aedes vexans* (Meigen), *Ochlerotatus triseriatus* (Say), *Anopheles punctipennis* (Say), *Ochlerotatus trivittatus* (Coquillett), *Anopheles walkeri* Theobald, *Ochlerotatus stimulans* (Walker), *Anopheles quadrimaculatus* (Theobald), and *Ochlerotatus canadensis* (Theobald) [12]. These thirteen species have been routinely collected and identified throughout the province of Ontario since 2002.

Ontario is Canada’s most populous province with approximately 20% of Canada’s population located in a few municipalities in southern Ontario [13], highlighting the importance of studying mosquito diversity and arbovirus transmission across the urban-rural ecological gradient. This region also experiences higher than average temperatures in the summer, which may contribute to WNV transmission by shortening the extrinsic incubation period of the virus in the mosquito vector [11,14].

Historically, Wood et al. [15] and Darsie and Ward [16] published species distribution maps of Ontario mosquito species but these maps do not indicate local species abundance or seasonal distribution. Knowledge of temporal and geospatial distribution of WNV vector species is crucial to the efficient collection of mosquitoes for future studies and arbovirus surveillance efforts. These data have the potential to contribute to a more effective larvicide program that utilizes established patterns of mosquito activity to target specific species at certain times of the year. Identifying high-risk regions of WNV vector activity may also contribute to more efficient and localized arbovirus awareness campaigns to alert the public in a time-sensitive manner. Additionally, many of these species have been implicated in other disease transmission cycles. With the threat of exotic viruses such as Zika virus and Chikungunya virus spreading across North America knowledge of mosquito vector distributions have never been more relevant.

Here we report spatial and temporal distribution estimates for WNV vector species derived from over a decade of mosquito surveillance data. In addition, we set out to investigate whether landscape variables could be used to enhance our prediction surfaces.

## 2. Materials and Methods

**Figure 1:**
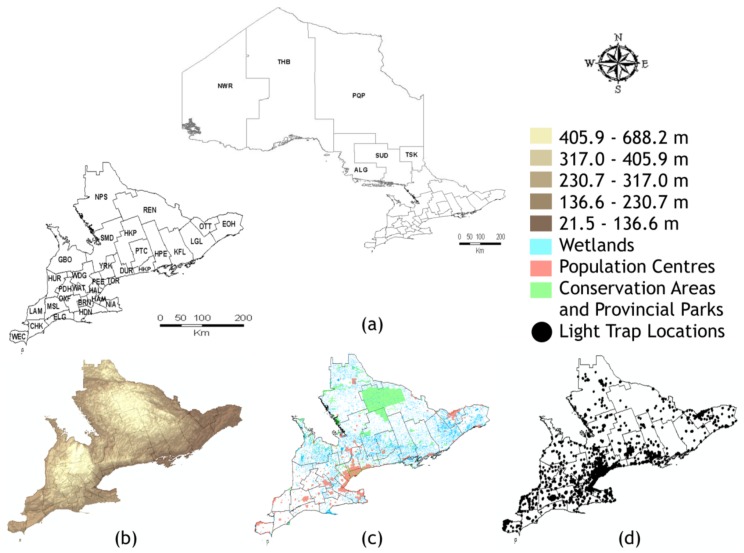
(**a**) Northern and southern Ontario, Canada with HU boundaries (solid black lines); (**b**) digital elevation model of southern Ontario; (**c**) map of conservation areas and provincial parks, wetlands, and population centres; (**d**) light trap locations in southern Ontario. Maps prepared in ArcGIS 10.4. Abbreviations: ALG, Algoma District; BRN, Brant County; CHK, Chatham-Kent; DUR, Durham Region; ELG, Elgin-St. Thomas; EOH, Eastern Ontario; GBO, Grey Bruce; HAL, Halton Region; HAM, City of Hamilton; HDN, Haldimand-Norfolk; HKP, Haliburton-Kawartha-Pine Ridge District; HPE, Hastings and Prince Edward Counties; HUR, Huron County; KFL, Kingston-Frontenac and Lennox & Addington; LAM, Lambton County; LGL, Leeds-Grenville and Lanark District; MSL, Middlesex-London; NIA, Niagara Region; NPS, North Bay Parry Sound District; NWR, Northwestern; OTT, City of Ottawa; OXF, Oxford County; PDH, Perth District; PEE, Peel Region; PQP, Porcupine; PTC, Peterborough County-City; REN, Renfrew County and District; SMD, Simcoe Muskoka District; SUD, Sudbury and District; THB, Thunder Bay District; TOR, City of Toronto; TSK, Timiskaming; WAT, Region of Waterloo; WDG, Wellington-Dufferin-Guelph; WEC, Windsor-Essex County; YRK, York Region.

Ontario has an area of 1.076 million km^2^ and is composed of 36 HUs (Figure 1). Algoma District (ALG), Northwestern (NWR), Thunder Bay District (THB), Porcupine (PQP), Sudbury and District (SUD), and the Timiskaming (TSK) HUs are known as the northern Ontario HUs; the remaining 30 HUs make up southern Ontario (Figure 1a). ArcMap version 10.4 (ESRI, Redlands, CA, USA) was used for general mapping purposes. We obtained the Ontario HU boundary file from Statistics Canada [17].

We obtained additional geographic database layers to describe the landscape of southern Ontario in more detail. We acquired the provincial digital elevation model (DEM) from the Ontario Ministry of Natural Resources and Forestry (MNR) [18] (Figure 1b); population centres digital boundary file from the 2016 census [19], population centres were defined as having at least 1000 individuals and a population density greater than or equal to 400 persons per square kilometer [20]; mapped wetland units from the MNR [21], wetlands were defined as both permanently or seasonally flooded lands where the water table is near the surface (e.g., marshes, swamps, bogs and in some cases shallow ponds or lakes); and a map of conservation areas and provincial parks (protected lands in Ontario) from the Land Information Ontario database [22] (Figure 1c). Ontario’s most populous HUs are Durham Region (DUR), Halton Region (HAL), City of Hamilton (HAM), City of Ottawa (OTT), Peel Region (PEE), City of Toronto (TOR), Windsor-Essex County (WEC), and York Region (YRK), most of which are located along the south-western edge of Lake Ontario (an area commonly referred to as the ‘Golden Horseshoe’). Conservation areas and provincial parks are scattered throughout Ontario. The largest provincial park (Algonquin Park) is in Renfrew County and District (REN) and extends into Haliburton-Kawartha-Pine Ridge District (HKP) (Figure 1c). Wetlands were least abundant in the south-western HUs and in PEE and TOR.

Each week from May to October, Centres for Disease Control and Prevention (CDC) miniature light traps (baited with dry ice) are set throughout Ontario as part of a province-wide mosquito surveillance program. Each HU manages their own surveillance program; the number of trapping nights and CDC miniature light traps set in each HU is not equal due to variable funding models among the HUs. CDC miniature light trap locations are presented in Figure 1d and the total number of trapping nights in each of the 36 HUs is presented in Table A1. Light traps are collected 24 h later and their contents sent to PHAC certified laboratories for species identification and diagnostic testing. Thousands of mosquitoes are collected each week, but only female WNV vector species are identified morphologically using the keys of Wood et al. [15] and Thielman and Hunter [23]; molecular identification is not required by PHO. Female mosquitoes are sorted by species into pools of no more than 50 specimens. Each week surveillance data are sent to PHO and published online as weekly surveillance reports [24]. We had been granted access by PHO officials to Ontario’s mosquito surveillance database for 2002 to 2014 including the collection date, global positioning system (GPS) coordinates, and species counts. Collection dates have been aligned to the epidemiological week (epi-week) calendar set out by the CDC. Additional data from the first three years of mosquito surveillance in Ontario (2002 to 2004) were provided by Entomogen Inc. (St. Catharines, ON, Canada).

**Figure A1:**
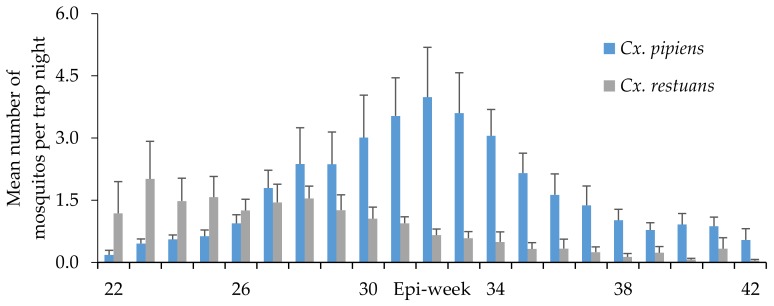
Seasonal distributions of *Cx. pipiens* and *Cx. restuans* derived from data collected during 2002 to 2007. Error bars represent the standard error.

Due to difficulties in correctly identifying *Cx. pipiens* and *Cx. restuans* morphologically PHO has required combining these species into a single pool for testing that we refer to as *Cx. pipiens*/*restuans* pools. We prepared seasonal distributions for *Cx. pipiens* and *Cx. restuans* from individual collection data obtained between 2002 and 2007, before PHO guidelines dictated they be combined (Figure A1). During the 2002 season, the first year of the surveillance program, light traps were not set in Grey Bruce (GBO), Huron County (HUR), Kingston-Frontenac and Lennox & Addington (KFL), Northwestern (NWR), Porcupine (PQP), Sudbury and District (SUD), Thunder Bay District (THB), and Timiskaming (TSK). Specimens of the *An. quadrimaculatus* species complex were not identified any further than *An. quadrimaculatus sensu lato*.

Statistical analyses were completed in Microsoft Excel 2010 with the Data Analysis Toolbox (Microsoft, Redmond, WA, USA), in ArcGIS 10.4 with the Spatial Analyst Toolbox, and with R version 3.4.2. [25]. The GPS locations, HU label, and number of collected WNV vectors were recorded for every light trap set from epi-week 21 to 42 (May to October) each year from 2002 to 2014. The total number of trapping nights was obtained for each light trap, epi-week, and HU. To account for sampling bias resulting from unequal trapping efforts among the HUs we calculated mean number of mosquitoes per trap-night (MMTN) for each individual CDC miniature light trap, epi-week, and HU over the 13 years. Weekly abundance data (from all 36 HUs) were plotted with the calculated standard error.

Our geospatial analyses are restricted to the 30 southern HUs due to sampling bias from individual light traps being separated more than 50 km in the northern HUs. GPS coordinates of each trap location containing MMTN data for each species was used for zonal statistical analysis. The average elevation within 10 km of each trap was identified. We performed a multiple ring buffer of 5 km increments up to 100 km around conservation areas and provincial parks, wetlands, and population centres. Daily flights of mosquitoes to search for shelter, mates, oviposition sites, blood, and nectar are typically short, 1–5 km [26]. Generated buffer layers were spatially intersected with trap locations to identify the proximity of traps to conservation areas and provincial parks, wetlands, and population centres.

Spatial autocorrelation of MMTN data was assessed using the Spatial Analyst Toolbox (ESRI, Redlands, CA, USA) to test MMTN data for spatial autocorrelation. We used Global Moran’s index (Gi) and local indicators of spatial association (LISA) to measure the degree of spatial autocorrelation for each species. We selected a zone of indifference weighting for our Gi calculations and LISA analyses. This method assigns points within a specified search radius a weighting of 1.0. Any points located outside of the search radius are weighted from 0.9 (closest to the search radius) to 0.0 (farthest from the search radius) according to a Gaussian distribution. Gi, z-score, and *p*-value were recorded with 5, 10, 15, 20 km lag periods. Gi evaluates the entire data set and assigns a value ranging from −1 to +1. There were three possible outcomes with the data set being dispersed (−1 < Gi < 0), randomly distributed (Gi = 0), or clustered (0 < Gi < +1). Significance was evaluated at *p* < 0.05 for spatial autocorrelation analyses [27].

Only MMTN data sets that exhibited significant Gi results were subjected to a LISA analysis. The lag distance with the largest significant Gi for each species was selected as the bandwidth. The local Moran’s index was recorded for each trap location. Point locations that were found to be statistically significant in the LISA analysis (*p* < 0.05) with a local Moran’s index greater than zero indicate clustering and were assigned as high-high (HH) if they occurred near other locations of high mosquito abundance or low-low (LL) if they occurred near surrounding locations of low mosquito abundance. Significant point locations with a local Moran’s index less than zero indicate outliers and were assigned as high-low (HL) if they are a high valued point surrounded by low values or low-high (LH) if they are a low valued point surrounded by high values [27]. Point locations with a *p*-value greater than 0.05 were assigned as not significant (NS).

Variograms were produced to illustrate spatial dependence among MMTN data and landscape variables using the gstat package (version 1.1–5) for R software (R Foundation for Statistical Computing, Vienna, Austria) [28,29]. We performed a qualitative analysis which consisted of a visual inspection of each individual variogram. Variograms identified as having strong spatial autocorrelation with MMTN data (i.e., resembles the standard variogram) were used to generate predicted mosquito abundance layers using the ArcMap Geostatistical Analyst extension. The kriging method (universal versus simple) was chosen based on the lowest error output from interpolated results. The following prediction errors were recorded for each prediction model: Root mean square standardized (RMSS), mean standardized (MS), root mean square (RMS), and average standard error (ASE). We proceeded with interpolation if relatively minimal prediction errors were observed with RMSS approximately equal to 1, MS approximately equal to 0, and RMS approximately equal to ASE [30]. For each species the prediction surface with the lowest error output and calculated standard error surface were clipped to the Ontario HU boundary file.

Principal components analysis was completed to explore correlations of MMTN data with spatially associated landscape variables using R software [21]. A scatter plot of the first and second principal components was generated for each species using the ggbiplot package (version 0.55) for R software [31]. Principal components analysis was used as a preliminary multivariate assessment of whether mosquito density was correlated with the landscape conditions (elevation, proximity to conservation areas and provincial parks, wetlands, and population centres).

## 3. Results

### 3.1. Analyses Including All 36 HUs

#### Seasonal Distribution of WNV Vectors in Ontario

**Figure 2:**
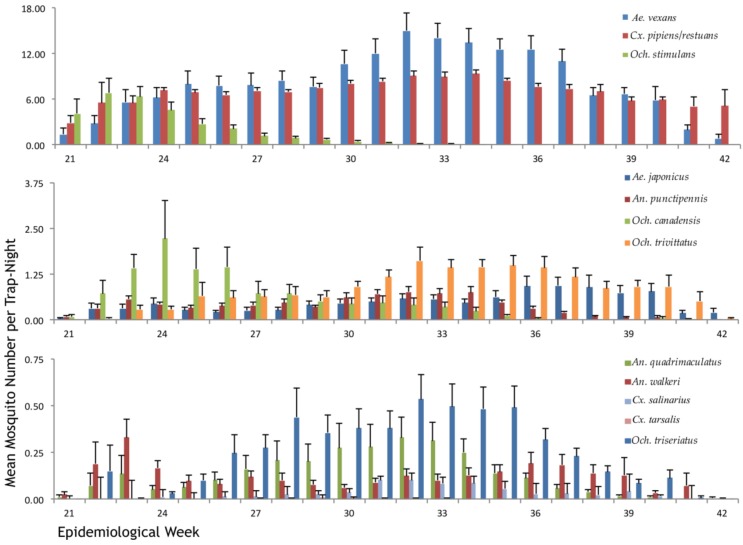
Seasonal distribution of 13 WNV (West Nile virus) vectors collected in Ontario, Canada from 2002 to 2014. Errors bars represent the standard error.

From 2002 to 2014 a total of 1,756,997 WNV vectors were identified which included 837,160 *Ae. vexans* (47.65%), 610,454 *Cx. pipiens*/*restuans* (34.74%), 82,045 *Och. trivittatus* (4.67%), 68,669 *Och. stimulans* (3.91%), 42,416 *Ae. japonicus* (2.42%), 35,201 *Och. canadensis* (2.00%), 34,260 *An. punctipennis* (1.95%), 23,426 *Och. triseriatus* (1.33%), 10,729 *An. quadrimaculatus* (0.61%), 9565 *An. walkeri* (0.54%), 2751 *Cx. salinarius* (0.16%), and 321 *Cx. tarsalis* (0.02%). Seasonal distribution of each vector species is presented in Figure 2. In general, our results indicate that mosquito populations in Ontario slowly increased from May to July and declined from August to October, except for *Och. stimulans* and *Och. canadensis*, which peaked in late May to early June and began to decline slowly after that. *Cx. pipiens* and *Cx. restuans* seasonal distributions are presented in Figure A1. *Cx. pipiens* was more abundant than *Cx. restuans*; *Cx. restuans* populations peaked early in May and begin to decline after that while *Cx. pipiens* abundance was the highest in August. These data were obtained between 2002 and 2007.

### 3.2. Geospatial Analyses of the 30 Southern Ontario HUs

#### 3.2.1. Exploratory Data Analysis

Our analysis, that did not include landscape variables, indicated weak positive spatial autocorrelation for the *Ae. vexans* spatial distribution (Table 1). All other species showed no spatial autocorrelation, suggesting their distribution in southern Ontario is statistically random and not clustered without incorporating additional landscape variables. No significant results were obtained for *An. punctipennis, An. walkeri*, and *Cx. tarsalis*, and these data sets were omitted from LISA analysis.

**Figure 3:**
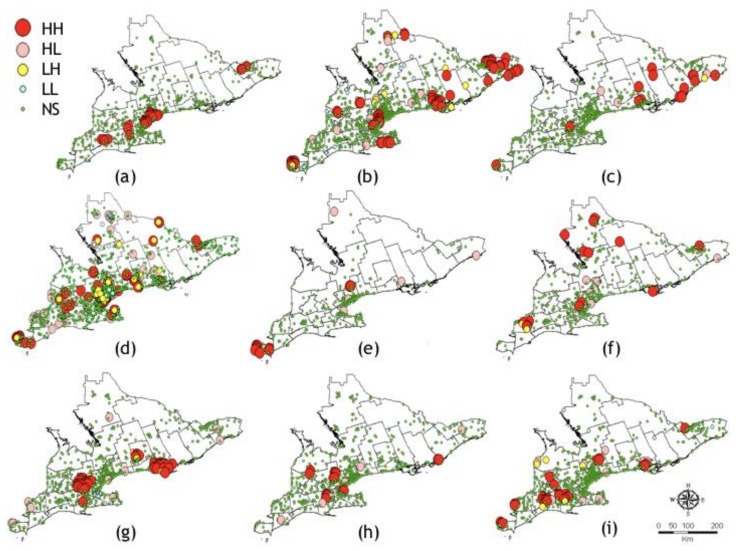
LISA cluster analysis of MMTN data. (**a**) *Ae. japonicus*; (**b**) *Ae. vexans*; (**c**) *An. quadrimaculatus*; (**d**) *Cx. pipiens/restuans*; (**e**) *Cx. salinarius*; (**f**) *Och. canadensis*; (**g**) *Och. stimulans*; (**h**) *Och. triseriatus*; (**i**) *Och. trivittatus*. HU boundaries are shown with a solid black line. Abbreviations, HH, high–high; HL, high–low; LH, low–high; LL, low–low; NS, not significant.

LISA cluster analysis of each statistically significant data set identified in Table 1 is presented in Figure 3. LISA cluster analysis identified 30 HH, 1 HL, 4 LH for *Ae. japonicus* (*n* = 638); 73 HH, 10 HL, 18 LH, and 31 LL trap locations for *Ae. vexans* (*n* = 995); 14 HH, 3 HL, 3 LH, and 2 LL for *An. quadrimaculatus* (*n* = 605); 145 HH, 71 HL, 31 LH, and 26 LL for *Cx. pipiens*/*restuans* (*n* = 3520); *Cx. salinarius*: 10 HH, 5 HL, and 2 LH (*n* = 272); 14 HH, 5 HL, and 4 LH for *Och. canadensis* (*n* = 443); 42 HH, 10 HL, 10 LH, and 11 LL for *Och. stimulans* (*n* = 707); 15 HH, 6 HL, and 1 LH for *Och. triseriatus* (*n* = 616); and 30 HH, 9 HL, 7 LH, and 5 LL for *Och. trivittatus* (*n* = 736) (Figure 3).

**Figure 4:**
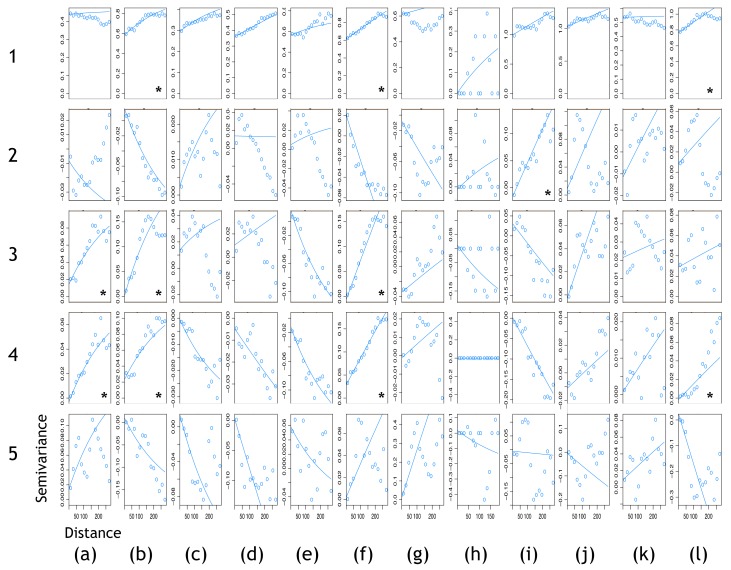
Cross-variography. (**a**) *Ae. japonicus*; (**b**) *Ae. vexans*; (**c**) *An. punctipennis*; (**d**) *An. quadrimaculatus*; (**e**) *An. walkeri*; (**f**) *Cx. pipiens/restuans*; (**g**) *Cx. salinarius*; (**h**) *Cx. tarsalis*; (**i**) *Och. canadensis*; (**j**) *Och. stimulans*; (**k**) *Och. triseriatus*; (**l**) *Och. trivittatus*. 1—MMTN; 2—MMTN, DEM; 3—MMTN, proximity to population centres; 4—MMTN, proximity to wetlands; 5—MMTN, proximity to conservation regions and provincial parks. * Indicates a spatially autocorrelated data set to be further explored in ArcMap 10.4 Geostatistical Analyst. Abbreviations: DEM, digital elevation model; MMTN, mean number of mosquitoes per trap-night; PARK, proximity to conservation areas and provincial parks; PC, principal component; POP, proximity to population centres; WET, proximity to wetlands; var., variance.

We generated cross-variograms of MMTN data against each landscape variable to determine whether individual or combinations of landscape variables can be used to strengthen previous assessments of spatial autocorrelation (Figure 4). Strong spatial autocorrelation was detected using individual landscape variables. Elevation (DEM) was identified as a key driver of *Och. canadensis* spatial distributions. Proximity to population centres was identified as a key driver of *Ae. japonicus*, *Ae. vexans*, and *Cx. pipiens*/*restuans* spatial distributions. Proximity to wetlands was identified as a key driver of *Ae. japonicus*, *Ae. vexans*, *Cx. pipiens*/*restuans*, and *Och. trivittatus* spatial distributions. Weak spatial autocorrelation was detected using MMTN data for *Ae. vexans, Cx. pipiens*/*restuans*, and *Och. trivittatus* spatial distributions.

**Figure A2:**
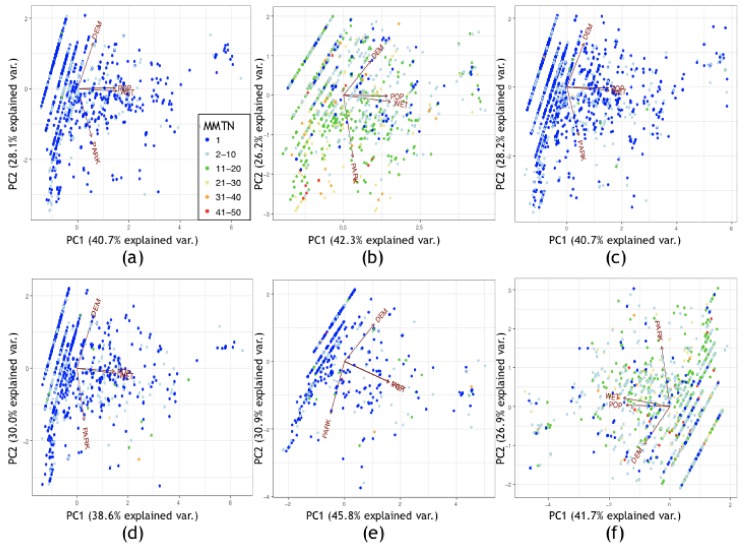

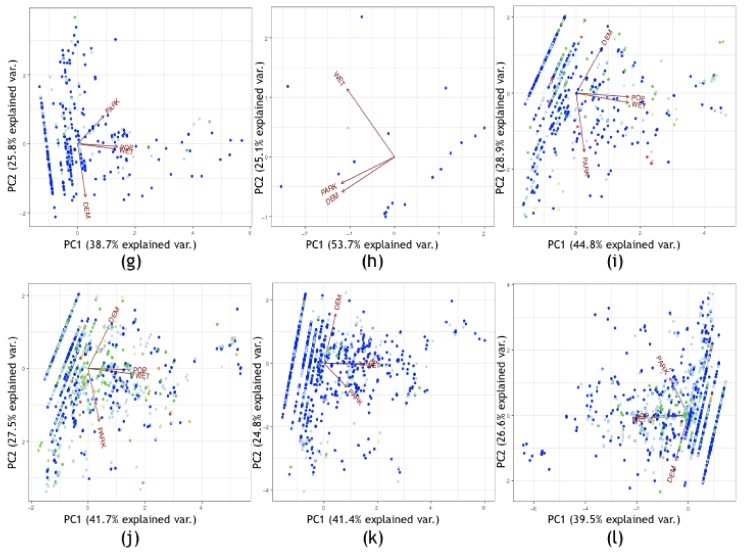
Ordination plots of the first 2 principal components. (**a**) *Ae. japonicus*; (**b**) *Ae. vexans*; (**c**) *An. punctipennis*; (**d**) *An. quadrimaculatus*; (**e**) *An. walkeri*; (**f**) *Cx. pipiens/restuans*; (**g**) *Cx. salinarius*; (**h**) *Cx. tarsalis*; (**i**) *Och. canadensis*; (**j**) *Och. stimulans*; (**k**) *Och. triseriatus*; (**l**) *Och. trivittatus*. Abbreviations: DEM, digital elevation model; PARK, proximity to conservation areas and provincial parks; PC, principal component; POP; proximity to population centres; WET, proximity to wetlands; var., variance.

We performed a principal components analysis of MMTN data against all landscape variables to determine whether a multivariate analysis (i.e., utilizing multiple landscape variables for prediction surface interpolation) can be used to refine predictions of mosquito species spatial distributions. Ordination plots of the first two principal components can be viewed in Figure A2. The principal component scatter plots show random scatter when incorporating all landscape properties together for every species which indicates that accurate prediction surfaces cannot be generated by combining two or more landscape variable data sets.

#### 3.2.2. Kriging/Co-Kriging

Each data set identified in Figure 4 as having strong spatial autocorrelation was used to produce the optimal kriged or co-kriged predicted MMTN and associated prediction error layers. A summary of the prediction errors is shown in Table 2. *Ae. vexans* and *Cx. pipiens*/*restuans* showed improved prediction surfaces (characterized by stronger prediction error parameters) by co-kriging with a landscape variable. Co-kriging MMTN and proximity to population centres data for *Och. trivittatus* had no benefit (i.e., identical prediction errors) when compared to universal kriging of the MMTN data alone (Table 2).

**Figure 5:**
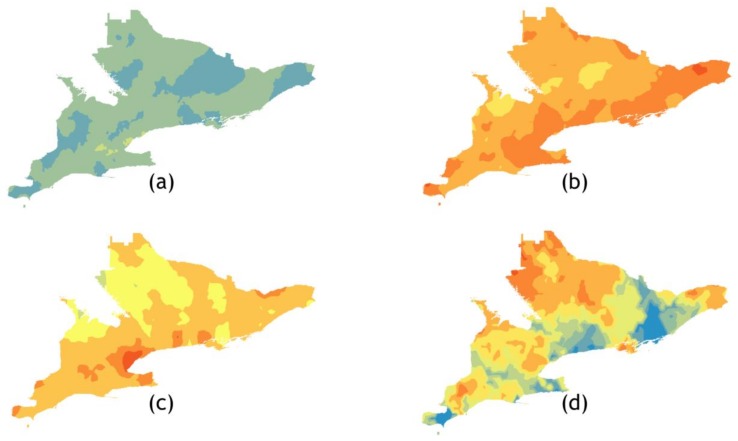

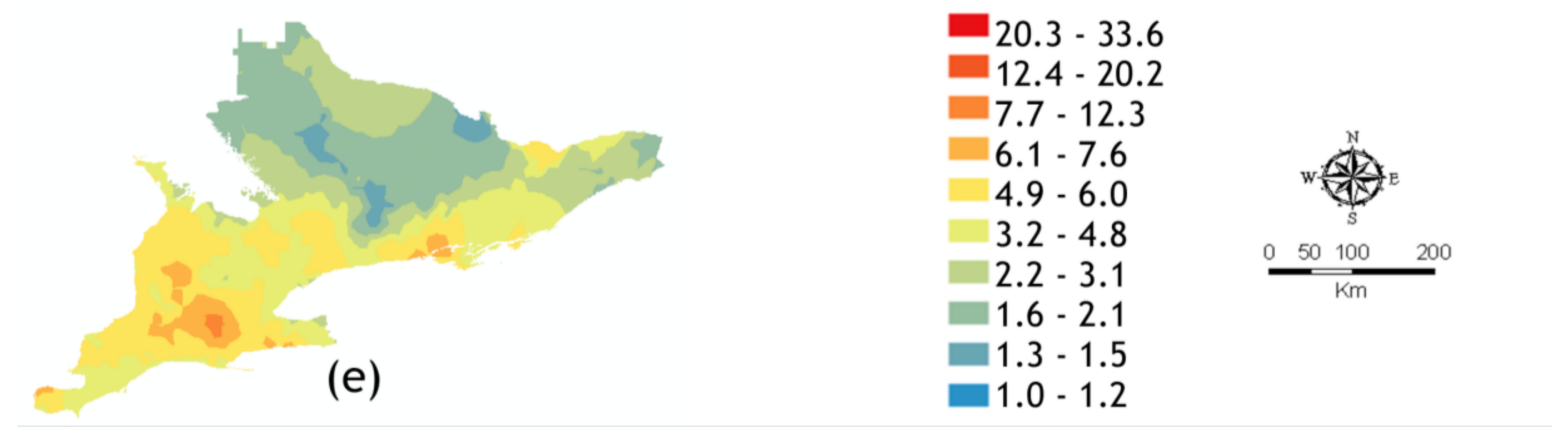
Predicted mean number of mosquitoes per trap-night data. (**a**) *Ae. japonicus* (simple kriging: MMTN, WET); (**b**) *Ae. vexans* (simple kriging: MMTN, POP); (**c**) *Cx. pipiens*/*restuans* (simple kriging: MMTN, WET); (**d**) *Och. canadensis* (universal kriging: MMTN, DEM); (**e**) *Och. trivittatus* (universal kriging: MMTN, POP). Abbreviations: DEM, digital elevation model; MMTN, mean number of mosquitos per trap-night; POP; proximity to population centres; WET, proximity to wetlands.

**Figure 6:**
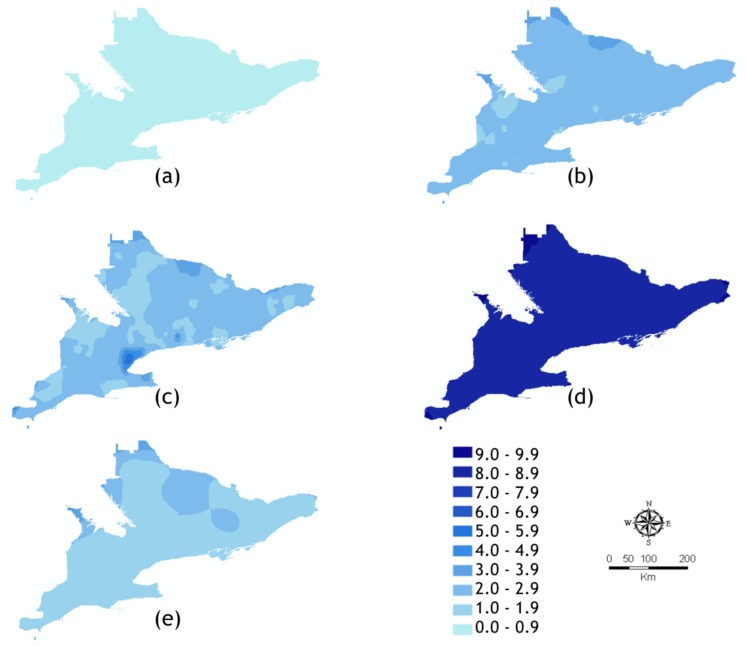
Standard error of prediction surfaces. (**a**) *Ae. japonicus* (simple kriging: MMTN, WET); (**b**) *Ae. vexans* (simple kriging: MMTN, POP); (**c**) *Cx. pipiens*/*restuans* (simple kriging: MMTN, WET); (**d**) *Och. canadensis* (universal kriging: MMTN, DEM); (**e**) *Och. trivittatus* (universal kriging: MMTN, POP). Abbreviations: DEM, digital elevation model; MMTN, mean number of mosquitos per trap-night; POP; proximity to population centres; WET, proximity to wetlands.

For each data set identified in Table 2 we present the optimal kriged or co-kriged predicted MMTN and the calculated standard error (Figure 5 and Figure 6). The highest predicted mosquito abundances were for *Ae. vexans* and *Cx. pipiens*, which was expected given the results from our seasonal distribution analysis. *Ae. vexans* showed moderate spatial clustering in Eastern Ontario (EOH), HAL, Haldimand-Norfolk (HDN), Hastings and Prince Edward Counties (HPE), and WEC. *Cx. pipiens*/*restuans* showed moderate clustering in the urban HUs of HAL, PEE, and TOR. *Och. canadensis* showed especially strong spatial clustering in the north region of North Bay Perry Sound (NPS) (Figure 5d). However, the *Och. canadensis* prediction surface also had the highest standard error (Figure 6d). The lowest predicted mosquito abundances were for *Ae. japonicus* which showed weak spatial clustering and low abundance throughout southern Ontario but had the lowest standard error among the co-kriged data sets (Figure 6a). *Och. trivittatus* showed moderate clustering in the south western HUs of Brant County (BRN), Oxford County (OXF), and Perth District (PDH). The error maps for *Ae. vexans*, *Cx. pipiens*/*restuans*, and *Och. trivittatus* showed low (+/−3.0 to 5.0) standard error.

## 4. Discussion

*Ae. japonicus* is an invasive species introduced to North America from Asia in the early 2000s [32] and was first detected in NIA in 2001 [33]. This species spread throughout most of southern Ontario in 4 years [33] and has been implicated as an efficient vector of WNV in laboratory studies conducted in the USA [9,10]. *Ae. japonicus* was collected significantly more in the urban HUs of HAL, PEE, TOR, and YRK, where its preferred oviposition sites, natural and artificial containers, are plentiful [33]. This species is collected throughout all of southern Ontario but low in abundance. This species has now been detected in all 36 HUs and has demonstrated its ability to thrive in both urban and rural habitats. Records in the published literature place this species as far west as British Columbia, Canada [34] and as far east as Newfoundland, Canada [35].

*Ae. vexans* has been well documented in Ontario for over 30 years. This species is a nuisance to humans and other large mammals, primarily due to the large populations that emerge [15]. This species has shown to be an efficient laboratory vector for WNV [9] and is also implicated in the transmission of dog heartworm (*Dirofilaria immitis*) [36]. Our analyses confirm that this species is highly abundant throughout the entire field season in Ontario. The kriged and LISA maps identified the highest mosquito densities in EOH, HAL, OTT, PEE, and WEC. *Ae. vexans* mosquitoes are known to travel far for food and breeding [15]. This floodwater mosquito prefers temporarily flooded areas and their abundance is known to correlate with weather conditions [15].

Three *Anopheles* species are monitored in Ontario for presence of WNV. The most abundant *Anopheles* species in Ontario is *An. punctipennis*. We are unable to comment on other members of *An. quadrimaculatus s.l.* as PHO does not require these species to be identified. Both *An. quadrimaculatus* and *An. punctipennis* are also known to transmit dog heartworm [36]. LISA cluster analysis revealed hot spots of *An. quadrimaculatus* activity in the eastern HUs of EOH, HKP, KFL, and LGL. *An. walkeri* used to be the most common *Anopheles* mosquito in Ontario [15] but its populations have been slowly declining over the past 30 years perhaps due to loss of habitat and global climate change. Larvae are typically found in pristine wetlands or ponds with high emergent vegetation (mostly cattails) and consistent water levels [15]. This is the only *Anopheles* species in Ontario known to overwinter as eggs. The eggs require long periods of cold conditioning to hatch, which is why this species is sensitive to climate change [15]. Given its preferred habitats we expected to observe positive spatial autocorrelation with abundance and proximity to wetlands; however, GI indicated no statically significant spatial distribution and each cross-variogram was unfit for spatial modelling, perhaps due to a lack of data or inadequate sampling methodologies (i.e., traps located too far from breeding sites).

In the current work, we present combined distribution data for *Cx. pipiens* and *Cx. restuans*. These species are very similar morphologically but do exhibit different host feeding preferences and seasonal and geographic distributions [15,16]. Historically, *Cx. pipiens* has been known to inhabit southern Ontario whilst *Cx. restuans* can be found throughout most of Ontario [15,16]; *Cx. restuans* populations peak in the spring whereas *Cx. pipiens* are most abundant in mid-summer [37,38] (Figure A1); *Cx. pipiens* are more abundant than *Cx. restuans* in Ontario (Figure A1); and *Cx. pipiens* are found more often near human dwellings [15]. *Cx. pipiens*’ greater abundance compared to *Cx. restuans* is likely to skew the data set; however, since *Cx. pipiens* and *Cx. restuans* collections are combined in Ontario we are unable to comment on each individual species or assess their individual involvement in arboviral transmission. *Cx. pipiens*/*restuans* pools test positive for WNV more than other any other species pool in Ontario but it is *Cx. pipiens*’ southern distribution, late summer population peaks, and attraction to human hosts near the end of the field season that make it more likely to transmit WNV to humans in Ontario [37,38,39,40]. Our MMTN prediction surface was similar to the predicted mean number of positive *Culex* mosquito pools generated by Giordano et al. [40]. This result was expected given that these species drive WNV epidemics in Ontario.

Contrary to Darsie and Ward [16], *Cx. salinarius* has been detected in Ontario since 2002. Wood et al. [15] also did not include this species in the list of species known to inhabit Ontario. However, it is likely that this species became established in Ontario due to a northern range expansion approximately 20 to 30 years ago [4]. We can confirm this species is now well established in the province of Ontario [4]. *Cx. salinarius* was collected throughout southern Ontario with the highest densities occurring in WEC. Wild *Cx. salinarius* have also been found to be naturally infected with dog heartworm, albeit in low numbers [36].

Historically *Cx. tarsalis* is rarely collected in Ontario [15]. A statistically significant surface prediction model was unable to be generated for this species due to a lack of data. Each year in Ontario a handful of specimens are collected and to date no species pools have tested positive for WNV [40], although they are a common WNV vector in the Western Provinces [41] and the United States [42]. This species drives WNV epidemics in the Western provinces of Canada and has also shown vector competency for Rift Valley fever virus in a laboratory setting [43]. Since this species is rarely collected in Ontario it is difficult to assess its role in WNV transmission. Repeated collections in rural HDN (data not shown) suggest a small population may be established here.

*Och. canadensis* and *Och. stimulans* are part of a group of species commonly referred to as ‘Spring *Aedes*/*Ochlerotatus*’. This common name is consistent with our observed seasonal distributions for these species. These are woodland pool mosquitoes, which, as the name suggests, overwinter as eggs laid in forest depressions that become filled with water during the spring ice melts [15]. Peak collections of *Och. canadensis* occurred in HAL, Lambton County (LAM), NPS, REN, and Wellington-Dufferin-Guelph (WDG) and for *Och. stimulans* in HPE, Region of Waterloo (WAT), and WDG. *Och. canadensis* has also been implicated in the transmission of eastern equine encephalitis [44]. To date, *Och. canadensis* species pools have not tested positive for WNV while only 2 *Och. stimulans* pools have tested positive [40].

*Och. triseriatus*, known as the eastern tree hole mosquito, prefers to oviposit in tree holes and artificial containers [15]. Hot-spots of *Och. triseriatus* activity were observed in BRN and WDG. *Och. trivittatus* was collected in large numbers in the (southwestern) HUs of BRN, LAM, Middlesex-London (MSL), OXF, PDH, WAT, and WDG. *Och. trivittatus* is known from a variety of larval aquatic habitats [15]. Both species are also known to be competent vectors for dog heartworm [36].

The LISA analysis presented here may be influenced by the unequal density of trapping locations in southern Ontario. Since this analysis used distances to establish neighbours the more populous HUs, such as those in the ‘Golden Horseshoe’, OTT, and WEC, which had higher spatial densities of traps in comparison to the other HUs (Figure 1d, Table A1), may influence the statistical analysis. Lower numbers of neighbours in some rural areas (or areas of lower trap density) could result in less statistical significance compared to the areas with higher trap densities. Despite the number of neighbours used to calculate values was highly variable, our results show clear differences in spatial clustering and associated statistical significance among species. For example, in contrast to *Ae. vexans* and *Cx. pipiens*/*restuans*, we observed strong spatial and statistically-significant clustering of *Och. canadensis* and *Och. trivittatus* that was not focused in highly populated urban locations. These results correspond with the mosquito prediction maps, which show low model errors in these regions for *Ae. japonicus*, *Ae. vexans*, and *Och. trivittatus*.

In the current work, we set out to determine whether prediction surfaces could be generated from data collected as part of the province-wide mosquito surveillance program and improved with the addition of landscape variables. We evaluated data sets using a multidisciplinary approach, which included geoprocessing of available landscape data, advanced geospatial statistical analyses, map interpolation, and ecological methods. Our analyses demonstrated that statistically significant prediction surfaces of mosquito abundance can be generated from existing regional data. Principal component analysis demonstrated that it was not suitable to use all landscape variables together to predict mosquito abundance. Variograms showing spatial autocorrelation between MMTN data with individual landscape variables provided evidence that we were able to incorporate the influence of each landscape variable on the spatial distribution of five species. MMTN data aggregated for *Ae. japonicus, Ae. vexans, Cx. pipiens*/*restuans, Och. canadensis*, and *Och. trivittatus*, showed strong spatial autocorrelation with individual landscape variables, and were interpolated using co-kriging methods. Based on the results of the co-kriging and standard error mapping, the analysis presented here is most useful for modeling the spatial distributions of *Ae. japonicus*, *Ae. vexans, Cx. pipiens*/*restuans and Och. trivittatus*. Proximity to landscape features, are generally consistent from year to year making them useful for prediction surface modelling and future work. However, it is likely that higher resolution and more refined spatial distribution of landscape characteristics would more effectively enhance models of mosquito abundance.

It is well established that mosquito abundance and seasonal distribution can vary from year to year due to changes in temperature, rainfall, and humidity [45,46,47]. Other factors such as locations of aquatic habitats, vegetative index, and land use and development have also been explored [48,49]. However, these studies were conducted on a much smaller scale when compared to the size of southern Ontario. The relative importance of these dynamic variables in driving mosquito spatial patterns at the regional scale was beyond the scope of research presented here. The utility of integrating refined remotely sensed land cover data products and regional models of dynamic seasonal meteorological conditions for modeling mosquito spatial patterns should be considered in future studies.

## 5. Conclusions

Knowledge of mosquito species abundance and seasonal distribution is crucial to developing a vector-borne disease response plan. Without records of vector species health officials would be unable to adequately assess the risk that a novel pathogen has of becoming established in Ontario, or whether local mosquito species might play a role in transmission. In the current work, we have identified when and where each WNV vector is abundant. Findings and approaches presented here are most useful for modeling the spatial distributions of *Ae. japonicus*, *Ae. vexans*, *Cx. pipiens*/*restuans*, and *Och. trivittatus*. This is key insight since we expect other container breeding exotic invasive species to share similar spatial distributions as *Ae. japonicus*; *Ae. vexans* is the most abundant WNV vector in Ontario; *Cx. pipiens*/*restuans* are competent vectors for WNV and test positive more than any other species pool; and *Och. canadensis* and *Och. trivittatus* are both vectors of dog heartworm. With these spatial models of mosquito density researchers and public health officials are better equipped to respond to the introduction of new viruses and mosquito species to Ontario. These data also have the potential to contribute to larvicide programs and public awareness campaigns. We recommend using local mosquito abundance to target specific species and warn the public in a time efficient manner. These data can also be used, in combination with our seasonal distribution data, to maximize efforts to collect each species for research or surveillance purposes. Recent outbreaks of Zika virus and Chikungunya in the southern United States underscore the value of utilizing mosquito spatial distributions in an effort to protect public health and arbovirus ecology in Ontario.

## Figures and Tables

**Table 1:** Spatial autocorrelation results for geospatial distribution of WNV vector abundance among the 30 southern Ontario HUs. Global Moran’s index values are presented with their *p*-value in brackets.

Species	5 km	10 km	15 km	20 km
*Ae. japonicus*	0.03 (0.386)	0.04 (0.109)	0.06 (0.010) ^1^	0.05 (0.014)
*Ae. vexans*	0.20 (<0.001) ^1^	0.20 (<0.001) ^1^	0.17 (<0.001) ^1^	0.17 (<0.001) ^1^
*An. punctipennis*	0.04 (0.207)	0.02 (0.506)	0.03 (0.521)	0.02 (0.351)
*An. quadrimaculatus*	0.04 (0.241)	0.06 (0.040) ^1^	0.04 (0.108)	0.07 (<0.001) ^1^
*An. walkeri*	0.00 (0.992)	0.04 (0.315)	0.04 (0.324)	0.03 (0.344)
*Cx. pipiens/restuans*	0.06 (0.030) ^1^	0.02 (0.389)	0.04 (0.058) ^1^	0.03 (0.131)
*Cx. salinarius*	0.10 (0.042) ^1^	0.08 (0.057) ^1^	0.05 (0.149)	0.05 (0.131)
*Cx. tarsalis*	−0.03 (0.401)	−0.03 (0.437)	−0.02 (0.423)	−0.02 (0.280)
*Och. canadensis*	0.07 (0.092) ^1^	0.09 (0.018) ^1^	0.11 (<0.001) ^1^	0.13 (<0.001) ^1^
*Och. stimulans*	0.12 (<0.001) ^1^	0.11 (<0.001) ^1^	0.11 (<0.001) ^1^	0.13 (<0.001) ^1^
*Och. triseriatus*	0.10 (0.005) ^1^	0.06 (0.075) ^1^	0.02 (0.420)	0.02 (0.250)
*Och. trivittatus*	0.11 (0.002) ^1^	0.13 (<0.001) ^1^	0.12 (<0.001) ^1^	0.10 (<0.001) ^1^

^1^ Indicates significant results (*p* < 0.05).

**Table 2:** Prediction error summary of kriged data sets. Abbreviations: MMTN, mean number of mosquitoes per trap-night; DEM, digital elevation model; POP; proximity to population centres; WET, proximity to wetlands; RMSS, root mean square standardized; MS, mean standardized; RMS, root mean square; ASE, average standard error.

Species	Variable(s)	Kriging Type	Model	RMSS	MS	RMS	ASE
*Ae. japonicus*	MMTN, POP	Universal	Stable	0.872	−0.005	2.153	2.470
	MMTN, WET ^1^	Simple	Stable	0.952	0.002	2.175	2.325
*Ae. vexans*	MMTN	Simple	Stable	0.984	0.019	8.521	8.645
	MMTN, POP ^1^	Simple	Stable	0.995	0.001	8.556	8.686
	MMTN, WET	Simple	Stable	1.018	−0.009	8.652	8.633
*Cx. pipiens/restuans*	MMTN	Universal	Stable	0.940	−0.001	9.880	10.629
	MMTN, POP	Simple	Spherical	1.034	−0.002	10.157	10.533
	MMTN, WET ^1^	Simple	Gaussian	1.012	−0.001	10.201	10.596
*Och. canadensis*	MMTN, DEM ^1^	Universal	Stable	0.942	0.003	9.050	9.644
*Och. trivittatus*	MMTN	Universal	Stable	1.121	−0.006	6.162	5.504
	MMTN, POP ^1^	Universal	Gaussian	1.121	−0.006	6.162	5.504

^1^ Indicates model used for prediction surface interpolation.

## Appendix A

**Table A1:** Total number of trapping nights in each HU by year.

HU	‘02	‘03	‘04	‘05	‘06	‘07	‘08	‘09	‘10	‘11	‘12	‘13	‘14
Algoma District	8	103	210	136	138	161	123	103	121	61	52	25	45
Brant County	76	47	139	120	117	105	139	150	123	117	98	143	114
Chatham-Kent	74	78	166	121	143	155	136	127	121	122	103	137	106
Durham Region	103	77	222	191	178	177	228	159	145	151	152	162	132
Elgin-St. Thomas	21	32	61	57	51	49	69	23	19	13	18	15	12
Eastern Ontario	64	46	100	119	104	115	119	76	66	73	70	92	92
Grey Bruce ^1^	0	41	89	89	36	8	1	2	0	9	1	0	0
Halton Region	160	336	302	354	318	335	347	192	185	175	228	212	208
City of Hamilton	104	138	611	572	504	575	530	488	516	463	344	371	418
Haldimand-Norfolk	35	43	214	227	211	255	230	185	100	37	20	41	33
Haliburton-Kawartha-Pine Ridge	32	86	74	343	299	292	316	123	98	36	117	136	166
Hastings and Prince Edward Counties	64	42	174	173	185	190	87	63	58	131	88	81	134
Huron County	0	85	94	114	128	122	131	130	122	103	53	74	106
Kingston-Frontenac and Lennox & Addington	0	34	35	33	33	25	27	24	63	46	50	41	53
Lambton County	40	66	122	158	185	168	179	124	134	128	131	126	115
Leegs-Grenville and Lanark District	63	41	105	103	100	98	98	44	39	29	45	138	100
Middlesex-London	172	175	221	209	241	368	400	277	360	379	327	351	378
Niagara region	115	149	224	189	185	162	151	246	274	308	295	364	350
North Bay Perry Sound District	5	89	124	120	127	131	131	71	81	66	69	47	91
Northwestern	0	14	74	56	105	90	61	46	44	21	19	26	61
City of Ottawa	10	417	481	532	462	419	436	419	338	307	299	427	476
Oxford County	114	49	144	137	97	122	125	115	114	123	121	148	132
Perth District	82	34	134	125	118	118	130	55	68	48	49	78	84
Peel Region	225	430	482	480	493	442	474	452	473	464	472	495	435
Porcupine	0	62	131	93	147	155	138	125	54	25	39	126	110
Peterborough County-City	24	28	70	75	73	63	76	50	46	54	67	78	71
Renfrew County and District	21	42	68	66	73	74	72	67	41	30	36	46	43
Simcoe Muskoka District	92	137	261	272	301	299	270	131	200	172	177	128	181
Sudbury and District	0	96	213	183	220	354	349	252	150	54	111	108	72
Thunder Bay District	0	60	162	154	36	73	47	18	32	38	17	26	16
City of Toronto	310	627	965	798	673	515	651	380	630	601	678	665	641
Timiskaming	0	63	51	57	41	63	63	63	47	34	27	36	77
Region of Waterloo	193	45	174	174	228	217	278	225	221	216	199	208	195
Wellington-Dufferin-Guelph	52	41	151	150	150	147	147	148	130	106	87	159	109
Windsor-Essex County	155	185	169	189	249	387	333	347	224	218	231	195	171
York Region	137	253	416	544	574	561	409	361	422	324	345	510	497

^1^ Grey Bruce largely reduced their surveillance program in 2008 after an internal risk assessment analysis deemed it no longer necessary.
